# The Effect of Virtual Reality on Emotional Response and Symptoms Provocation in Patients With OCD: A Systematic Review and Meta-Analysis

**DOI:** 10.3389/fpsyt.2021.733584

**Published:** 2022-02-01

**Authors:** Bahram Dehghan, Saied Saeidimehr, Mehdi Sayyah, Fakher Rahim

**Affiliations:** ^1^Department of Medicine, Naft Grand Hospital, The Health Affair Organization of Oils and Refineries Industry, Ahvaz, Iran; ^2^Department of Aging, University of Social Welfare and Rehabilitation Sciences, Tehran, Iran; ^3^Education Development Center (EDC), Ahvaz Jundishapur University of Medical Sciences, Ahvaz, Iran; ^4^Research Center of Thalassemia and Hemoglobinopathies, Health Research Institute, Ahvaz Jundishapur University of Medical Sciences, Ahvaz, Iran; ^5^Metabolomics and Genomics Research Center, Endocrinology and Metabolism Molecular-Cellular Sciences Institute, Tehran University of Medical Sciences, Tehran, Iran

**Keywords:** virtual reality (VR), obsessive-compulsive disorder (OCD), meta-analysis, emotional response generation, symptoms provocation

## Abstract

This systematic review and meta-analysis aimed to evaluate the effectiveness of virtual reality (VR)-based technology on emotional response and symptoms in patients with obsessive–compulsive disorder (OCD). We systematically searched major electronic databases, including PubMed/Medline, Scopus, Embase, ISI Web of Science, PsycINFO, and Cochrane central, up to April 14, 2021, with no data or language limits. We performed reference, related articles, and citation searches to find additional articles. We included original articles comparing and studying VR-based technology in patients with OCD against the control group. We observed that VR significantly increases in anxiety (SMD = 2.92; 95% CI 1.89–3.94, *p* < 0.0001; *I*^2^ = 95%), disgust (SMD = 2.52; 95% CI 1.36–3.68, *p* < 0.0001; *I*^2^ = 95%), urge to wash (SMD = 3.12; 95% CI 1.92–4.32, *p* < 0.0001; *I*^2^ = 94%), checking time (SMD = 1.06; 95% CI 0.71–1.4, *p* < 0.0001; *I*^2^ = 44%), number of checking behavior (SMD = 1.45; 95% CI 0.06–2.83, *p* = 0.04; *I*^2^ = 93%), and uncertainty (SMD = 2.59; 95% CI 0.90–4.27, *p* = 0.003; *I*^2^ = 70%) in OCD patients compared with healthy controls using a random-effect model. This meta-analysis found that this environment has a moderate enhancement in emotional response and symptoms test scores of patients with OCD. However, our findings should be generalized with caution due to the lack of standardized methods and high heterogeneity among included evidence. The appropriate mode of integrating VR-based technology for patients with OCD requires more exploration.

## Introduction

Obsessive–compulsive disorder (OCD) is a chronic anxiety disorder associated with excessive worries and mental preoccupation, distress, and low self-esteem. The lifetime prevalence of OCD was estimated as 2.3–3% (1, 2). OCD has two main core symptoms, including obsessions (annoying and repetitive thoughts and images) and compulsions (behaviors that must be carried out repeatedly) (3). This disorder occurs in both males and females, but it is more common among men than women (4). The exact etiology and cause of OCD are still unclear and often poorly recognized; however, it may be caused by a combination of genetics and childhood experiences (5, 6). Early detection of these disorders facilitates timely intervention to treat patients with OCD and help them to cope with the disease (7). Direct measurements of the main symptoms of OCD in the clinical office, especially using new technologies such as computer-based methods, may lead to more objective and comprehensive prevention and assessment of the disease (8).

The concept of a virtual environment (VE) has defined virtualization as the procedure by which a human viewer interprets a patterned sensory impression to be an extended object in an environment other than that in which it physically exists (9). The VE should integrate the contributor as part of the environment, so that head motions result in motion parallax from the participant's viewpoint, and a number of physiological and vestibular responses associated with focusing and object tracking are stimulated. This is a way of expressing the common view that presence is the sense of “being there” in the VE or similarly the sense of being in the place depicted by the virtual reality (VR) rather than in the real physical place where the participant's body is really located (9). Due to the remarkable capabilities of this technology, VR is used in various fields, especially in the medical field, in which real-world experience can be costly or risky (10). In the medicine and treatment of mental disorders, VR can overcome fears during behavioral therapy in order for the patient to face challenges in a controlled environment (11, 12). Given the diverse manifestations of the disease, OCD is often poorly recognized or hard to recognize; consequently, this problem leads to a significant delay between onset of OCD symptoms and initiation of effective treatment or treated appropriately (13). The reasons proposed in research include difficulties and shame in expressing the symptoms (7) or pronouncing symptoms mostly in private areas like home or office, not in a safe location such as the clinician's workplace (14). This will increase the chance of recall and interviewer bias. As claimed in recent studies, VR offers new possibilities to achieve a direct observation of the OCD symptoms, which may lead to a further objective and inclusive assessment of the disease (15). From the clinical point of view, recent lines of evidence showed that VR is able to concurrently provoke psychiatric symptoms (16). In OCD, numerous studies have considered the feasibility of VR as an assessment tool, mostly aiming to evoke fear of contamination and control behaviors. Objective behavioral indices have also been used to assess OCD-associated behavior at home and in the office to evaluate how persons cope with OCD-related tasks (15, 17–19).

VR-enhanced exposure therapy (VRET) is receiving greater attention and is considered as an interesting alternative for the traditional treatment, particularly in the field of anxiety disorders (20). VRET is being used in several ways to help individuals with OCD; a few sessions of traditional therapy or an entire VR-based therapy may be performed (21). Nevertheless, VRET offers a harmless and cost-effective alternative compared to traditional methods, in some conditions in which exposure is difficult or possibly risky such as driving phobia or when the cost of treatment is excessive like flight phobia (22, 23). So far, VRET has been used to assess and treat several mental disorders (24). Powers and Emmelkamp conducted a meta-analysis on 13 studies to assess VRET compared to *in vivo* exposure and control conditions in anxiety disorders and showed that VRET is superior to *in vivo* conditions (22). Mühlberger et al. conducted a randomized control trial (RCT) to assess the effect of repeated VRET on flight phobia and reported improved VRET group was greater than in the relaxation group (25). Mühlberger et al. also performed an RCT to evaluate the efficacy of one-session VRET on flight phobia and claimed that this therapy effectively reduced flight phobia for up to 1 year (26). Walshe et al. investigated the effectiveness of the combined use of game reality and VRET for the treatment of driving phobia post-accident even and suggested that this treatment may have a useful role in the treatment of driving phobia (27, 28).

In some studies, a VR environment was used to concurrently provoke and observe psychiatric symptoms (16, 29), while in other groups of studies, VR games could be considered as a further diagnostic capability to help medical experts detect OCD and accelerate the initiation of treatment (19, 30). To the best of our knowledge, systematic reviews and meta-analyses about the scientific evidence on the future use of VR in patients with OCD are limited. Therefore, this systematic review and meta-analysis aimed to evaluate the effectiveness of VR-based technology on emotional response and symptoms in patients with OCD.

## Methods

This meta-analysis was conducted in line with the Preferred Reporting Items for Systematic Reviews and Meta-Analyses (PRISMA) (31) and with meta-analysis of observational studies in epidemiology (MOOSE) (32) guidelines.

### Search Strategy

We systematically searched major electronic databases, including PubMed/Medline, Scopus, Embase, ISI Web of Science, PsycINFO, and Cochrane central using (“virtual reality” OR “virtual reality exposure therapy” OR “virtual systems” OR “augmented reality” OR “videogame” OR “video games” OR “exergames” OR “exergaming” OR “play-based therapy” OR “commercial games” OR “visualization technologies”) AND (“obsessive compulsive disorder” OR “obsessive-compulsive disorder” OR “OCD”) up to April 14, 2021, with no data or language limits. We performed reference, related articles, and citation searches to find additional articles.

### Formulating Research Question

We defined the research question of the systematic review clearly in terms of populations, interventions, comparators, outcomes, and study designs (PICOS) (Table 1).

**Table 1:** The description of populations, interventions, comparators, outcomes, and study designs (PICOS) used in this review.

**Population**	**Intervention**	**Comparison**	**Outcome**	**Study designs**
Patients with OCD Any ages	Virtual reality (VR)	**–**	**VR scoring** (break in time, break in choice, break in social rules, divided attention, self-correction, and preservation) **OCD symptoms** (anxiety, disgust, urge to wash, checking time, number of checking behavior, and uncertainty)	Original articles comparing and studying VR technology in patients with OCD against the control (case–control)

### Inclusion and Exclusion Criteria

We included original articles comparing and studying VR technology in patients with OCD against the control group. As a condition, all patients should be diagnosed by a clinical psychologist or psychiatrist meeting the Diagnostic and Statistical Manual of Mental Disorders (DSM-IV) criteria for OCD. Articles were excluded if provided no comparison, or the article type was letter, commentary, and case report, and if patient data overlapped with another included study.

### Data Extraction

Two authors (FR and SS) independently extracted data from included studies. The predefined data extraction form includes author's name, publication year, country, study design, study population, age of included patients, setting, and diagnosis methods of OCD. Any discrepancies were resolved either through discussion or re-checking the publication by the third reviewer until consensus was reached.

### Exposure

We divided the included studies intervention to 3D interactive models or VR simulations according to the Zhao et al. study (33). In this review, VR methods including types of VR environment, as non-invasive and invasive, could be performed as a single intervention or blended with others (34).

### Outcome Measures

We identified VR scoring parameters, including break in time, break in choice, break in social rules, divided attention, inefficiencies, self-correction, and preservation (35). In brief, breaks were classified in line with the descriptions, including break in time, which is defined as going to the shopping chart after 5 min; break in choice, which is described as buying two products instead of just one; and break in social rules, which is defined as going into a specific place and asking the examiner what to buy. Moreover, in case of partial task failures, the specific items are classified into divided attention (occurs when mental focus is directed toward multiple ideas, or tasks, at once), self-correction (occurs when Self-Administered Behavioral Skills-Based At-Home is directed toward tasks), and preservation (occurs when educating people around the world by creating and hosting virtual reality content to preserve historical sites) (36). We also assessed OCD symptoms in the VR environment using parameters of emotional responses expressing anxiety, disgust, tension, urge to wash, uncertainty, the number of checking behaviors, and duration of the checking performance.

### Assessment of Methodological Quality and Risk of Bias (ROB)

Two authors (FR and SS) independently performed the methodological quality assessment using the Newcastle-Ottawa Scale (NOS) (37). NOS scores were categorized into three groups, including very high risk of bias (0–3 NOS points), high risk of bias (4–6), and low risk of bias (7–9). The NOS scale has three components assessing studies on participant selection, comparability, and outcome/exposure assessment. A study is awarded stars for items within each category for a maximum of nine stars. We decided to rate studies as low risk of bias if they received nine stars, moderate risk of bias if they received seven or eight stars, and high risk of bias if they received less.

### Data Analysis

During the meta-analysis, the standardized mean difference (SMD) index with 95% confidence interval between the two groups for each study was calculated, and pooled SMD was observed and reported. Heterogeneity within and between studies was assessed using Cochrane's *Q*-statistics. The effect of heterogeneity was evaluated using *I*^2^ in the range between zero and 100. The values of *I*^2^ of more than 75, 50, and, 25 indicate high, medium, and low heterogeneity. For moderate to low heterogeneity, the fixed effect method was used, while for high heterogeneity, the random effect method was performed. We used the Funnel plot, Begg's correlation test, and Egger test to evaluate the potential source of publication bias (38). The analyses were performed using Revman 5.4.3. *p* < 0.05 was considered as a statistically significant level.

## Results

### Study Characteristics

**Figure 1:**
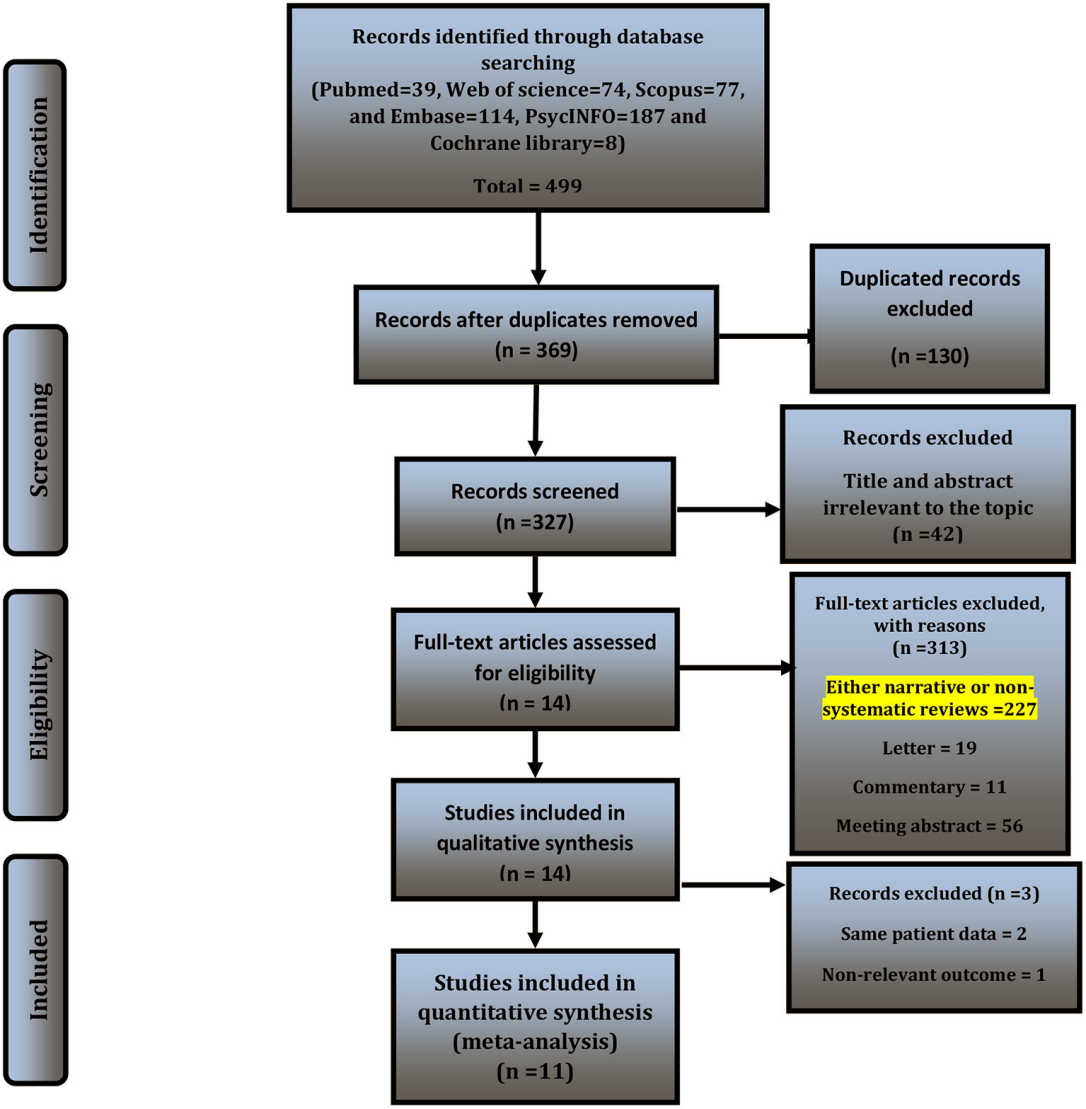
PRISMA flowchart of the study selection process.

The details of the study selection process were depicted in Figure 1. Overall, 499 studies were identified through initial search, of which 369 records remained and were screened. Of 369 records, 42 were excluded after the title and abstract screening. Then, we excluded 313 records due to eligibility, leaving 14 studies. Three studies were excluded, of which two (39, 40) included the same patient data as LaPaglia et al. (41), and one reported non-relevant outcome in terms of fMRI findings and brain activation signals (42). Finally, 11 records met the criteria and were included in the qualitative and qualitative analysis (Table 2) (15, 18, 19, 41, 43–49).

**Table 2:** Characteristics and results of included studies that report on the effectiveness of virtual reality on emotional and symptoms in patients with OCD.

**Study ID**	**Country**	**Population *n*/Age**	**Groups**		**Type of exposure**	**VR model**	**Key findings**	**Quality score**
			**Patient**	**Control**				
Inozu et al. (43)	Turkey	66/22.0 ± 2.43	OCD	OCD-c	Non-immersive	Computer-based HTC Vive VR system	VR can be used as an alternative exposure tool in the treatment of contamination-based OCD	5
van Bennekom et al. (15)	Netherlands	52/–	OCD	HC	Non-immersive	VR environment through a virtual version of the Multiple Errand Test (V-MET) using the NeuroVR software	VR game could help in assessing core OCD symptoms and recognizing OCD	8
McCabe-Bennett et al. (44)	Canada	76/34.3 ± 12.43	OCD	OCD-c	Non-immersive	Visual and auditory simulation (i.e., white room noise) with an Oculus Rift_ consumer-release version HMD, model number 301-00200-03	VR provided an opportunity to examine responses to increasing clutter levels	8
Jalal et al. (45)	Italy	29/26.7 ± 6.43	OCD	HC	Immersive	VR environment included visual and RHI	Using a fake hand during the RHI, instead, may provide a clever and convenient alternative	5
Pedroli et al. (46)	Italy	58/36.7 ± 12.73	OCD	HC	Non-immersive	NeuroVR (Version 2.0) a free software with a pre-existing virtual environment followed by NeuroVirtual 3D	Discriminating OCD patients from controls, with VR	8
van Bennekom et al. (19)	Netherlands	16/31.5 ± 7.9	OCD	HC	Non-immersive	VR environment included visual and RHI	VR is a potential valuable tool to objectify and standardize an OCD diagnosis	8
Laforest et al. (47)	Canada	32/30.5 ± 7.9	OCD	HC	Immersive	VR immersion system is made of six projected surfaces: four walls, the floor, and the ceiling all running Virtool VPPublisher Unlimited 5.0	Unwanted negative side effects induced by immersions in VR were higher in the OCD group	7
la Paglia et al. (41)	Italy	32/33.07 ± 9.09	OCD	HC	Immersive	VR environment through a V-MET using the NeuroVR software	VR is sensitive to evaluate the functional status of OCD patients with normal cognition	8
Cipresso et al. (48)	Italy	32/36.62 ± 11.09	OCD	HC	Immersive	VR environment through a V-MET using the NeuroVR software	VR allowed us to take into account deficits of volition and the relative dys-executive functions associated with OCD patients	8
Kim et al. (18)	Republic of Korea	70/29.52 ± 10.07	OCD	HC OCD-c	Non-immersive	VR with an IBM-compatible computer and an SVGA color HMD with a three-degrees-of-freedom tracker	Behavioral measures are able to improve the assessment of OCD by VR	7
Kim et al. (49)	Republic of Korea	63/34.52 ± 9.17	OCD	HC	Non-immersive	VR with an IBM-compatible computer and an SVGA color HMD with a three-degrees-of-freedom tracker	VR technology has a value as an anxiety-provoking or treatment tool for OCD	8

*VR, Virtual Reality; OCD, Obsessive–Compulsive Disorder; OCD-c, OCD-control; HC, Healthy controls; V-MET, virtual version of the Multiple Errand Test; HMD, head-mounted display, RHI, rubber hand illusion; SVGA, Supper Video Graphics Array; NOS scores categorized into three groups, including very high risk of bias (0–3 NOS points), high risk of bias (4–6), and low risk of bias (7–9)*.

### Participants and Study Characteristics

The final Selected Studies Encompassed a Sample of 315 Adults with OCD and 234 Healthy Controls. Of the Included Studies, Ten Records Presented the Mean Age of Participants Ranging from 22.0 (±2.43) to 36.7 (±12.73) Years.

### Quality of Study Assessment and Risk of Bias

The majority of the studies were rated as high quality (15, 18, 19, 41, 44, 46–49). Only **two** studies were rated as low quality in the assessment (43, 45) (Supplementary Table S1).

### VR Scoring

**Figure 2:**
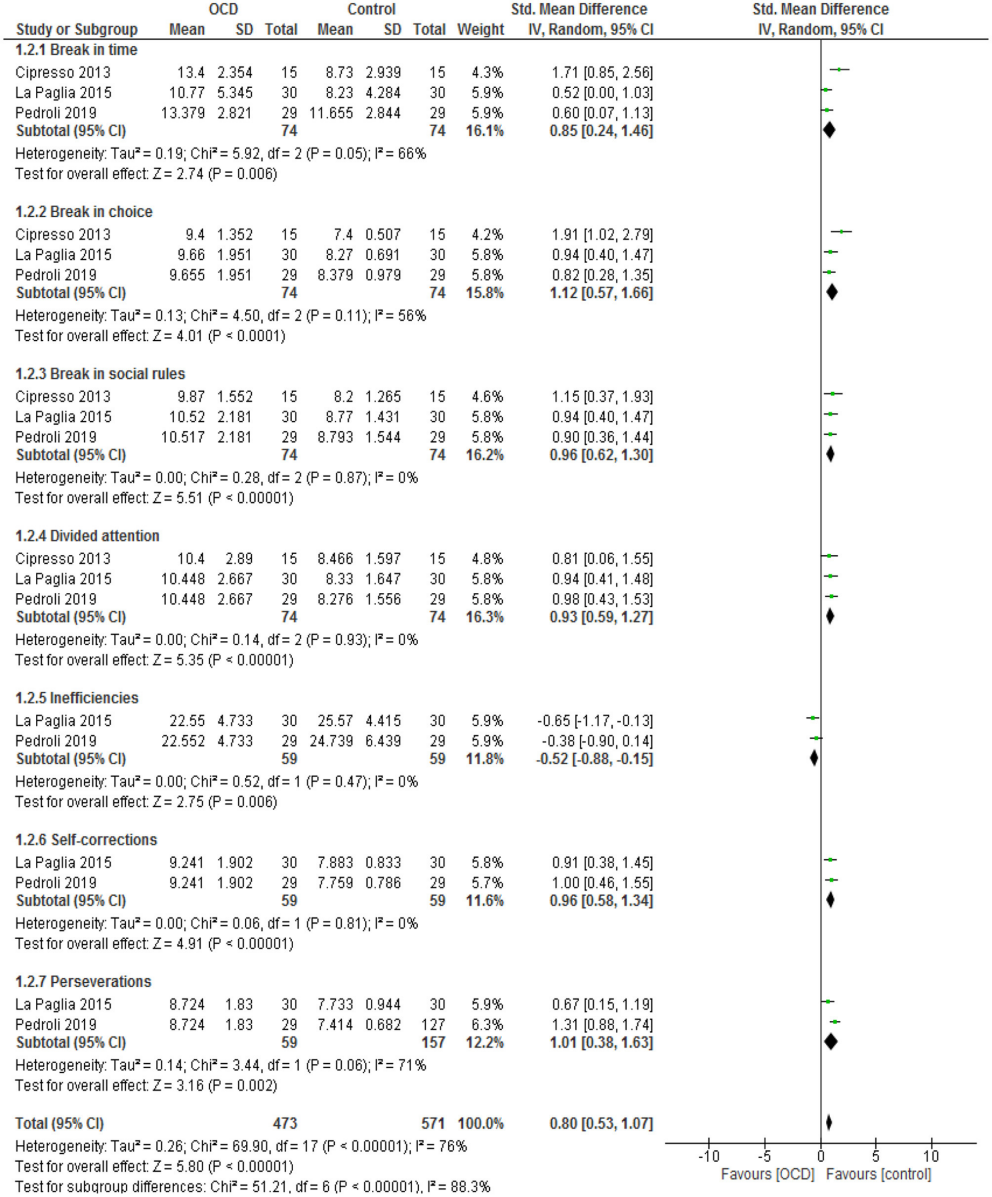
Forest plot for VR scoring. The green blocks indicate the weight assigned to the study, the horizontal line depicts the CI, and the black rhombus shows the overall result. IV, inverse variance; Std, standard.

We observed significant increase in break in time (SMD = 0.85; 95% CI 0.24–1.46, *p* = 0.006; *I*^2^ = 66%), break in choice (SMD = 1.12; 95% CI 0.57–1.66, *p* < 0.0001; *I*^2^ = 56%), break in social rules (SMD = 0.96; 95% CI 0.62–1.30, *p* < 0.0001; *I*^2^ = 0%), divided attention (SMD = 0.93; 95% CI 0.59–1.27, *p* < 0.0001; *I*^2^ = 0%), self-correction (SMD = 0.96; 95% CI 0.58–1.34, *p* < 0.0001; *I*^2^ = 0%), and preservation (SMD = 1.01; 95% CI 0.38–1.63, *p* = 0.002; *I*^2^ = 71%) in OCD patients compared with healthy controls using a random-effect model (Figure 2), while we identified a significant decrease in inefficiencies (SMD = −0.52; 95% CI −0.88 to −0.15, *p* = 0.006; *I*^2^ = 0%) in OCD patients compared with healthy controls using a random-effect model (Figure 2).

### OCD Symptoms

**Figure 3:**
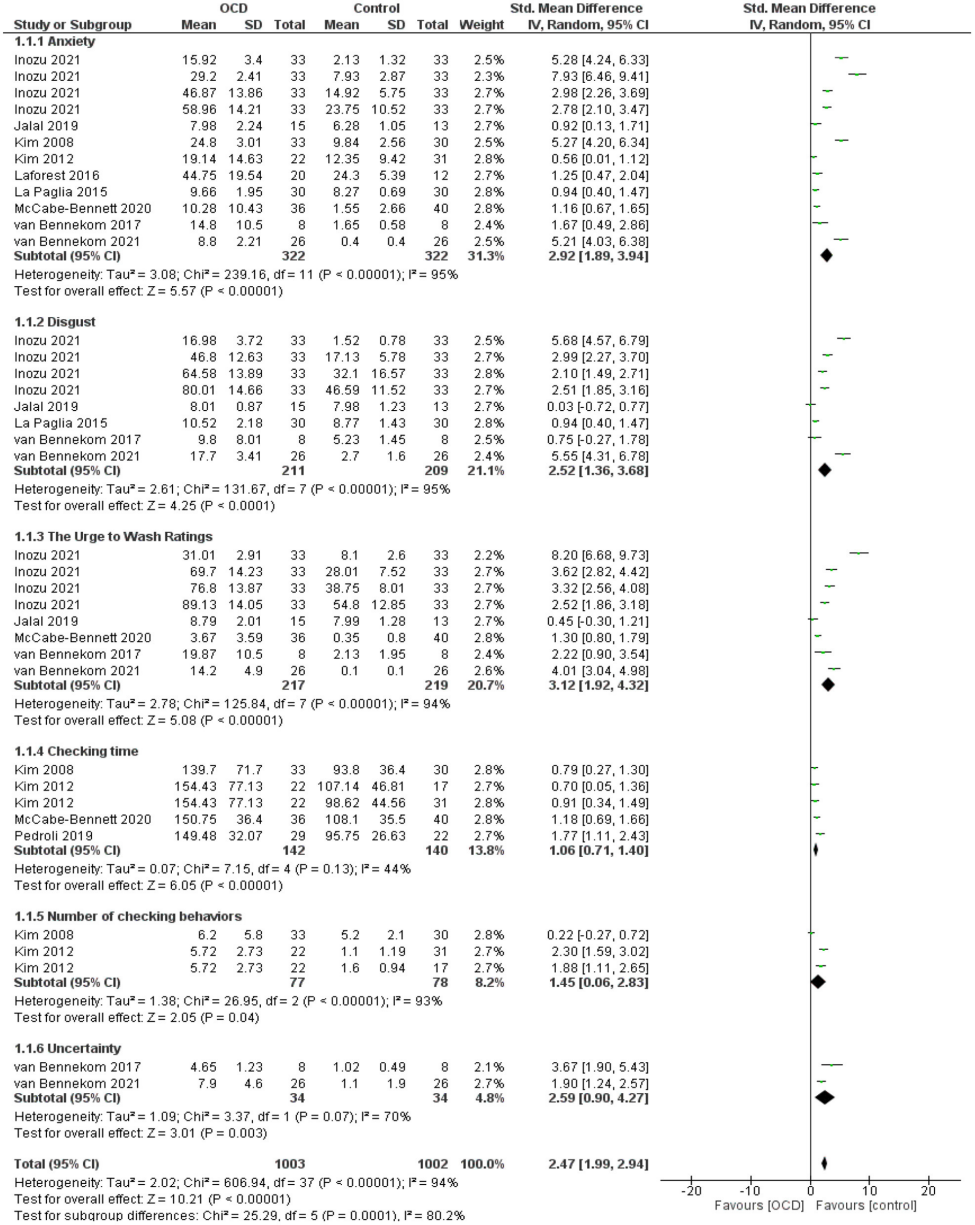
Forest plots for OCD symptoms. The green blocks indicate the weight assigned to the study, the horizontal line depicts the CI, and the black rhombus shows the overall result. IV, inverse variance; Std, standard.

We observed that the VR environment significantly increases in anxiety (SMD = 2.92; 95% CI 1.89–3.94, *p* < 0.0001; *I*^2^ = 95%), disgust (SMD = 2.52; 95% CI 1.36–3.68, *p* < 0.0001; *I*^2^ = 95%), urge to wash (SMD = 3.12; 95% CI 1.92–4.32, *p* < 0.0001; *I*^2^ = 94%), checking time (SMD = 1.06; 95% CI 0.71–1.4, *p* < 0.0001; *I*^2^ = 44%), number of checking behavior (SMD = 1.45; 95% CI 0.06–2.83, *p* = 0.04; *I*^2^ = 93%), and uncertainty (SMD = 2.59; 95% CI 0.90–4.27, *p* = 0.003; *I*^2^ = 70%) in OCD patients compared with healthy controls using a random-effect model (Figure 3).

We performed subgroup analysis according to the geographic region (Asia, Europe, and America), VR environments (non-immersive and immersive models), and comparators (OCD-control and Healthy control). Findings were heterogeneous according to the test results and led to using a random-effect model (Table 3). Moderator analyses showed a significant increase in anxiety, disgust, and urge to wash, in the Asian population, non-immersive VR environments, and compared to healthy controls. The rest of the potential moderators could not be analyzed due to an inadequate number of studies.

**Table 3:** Summary statistics for moderators.

**Subgroup**	***n***	**SMD**	**95%CI**	***P*-value**	***I^**2**^***
**Anxiety**
**Region**
Asian	6	4.07	2.22, 5.92	<0.0001	97%
Europe	4	2.13	0.47, 3.79	0.01	93%
America	2	1.19	0.77, 1.60	<0.0001	0%
**VR environments**					
Non-immersive	9	3.58	2.23, 4.94	<0.0001	96%
Immersive	3	1.01	0.62, 1.40	<0.0001	0%
**Comparator**					
HC	6	3.35	1.76, 4.94	<0.0001	97%
OCD-c	6	2.5	1.02, 3.98	0.0009	94%
**Disgust**					
**Region**					
Asian	4	3.24	2.04, 4.44	<0.0001	91%
Europe	4	1.75	−0.07, 3.57	0.06	95%
America	0	–	–	–	–
**VR environments**					
Non-immersive	6	3.21	1.98, 4.44	<0.0001	92%
Immersive	2	0.52	−0.37, 1.41	0.25	74%
**Comparator**					
HC	4	1.75	−0.07, 3.57	0.06	95%
OCD-c	4	3.24	2.04, 4.44	<0.0001	91%
**Urge of wash**					
**Region**					
Asian	4	4.27	2.52, 5.93	<0.0001	93%
Europe	3	2.21	−0.09, 4.52	0.06	94%
America	1	–	–	–	–
**VR environments**					
Non-immersive	7	3.51	2.27, 4.74	0.0001	94%
Immersive	1	–	–	–	–
**Comparator**					
HC	3	2.21	−0.09, 4.52	0.06	94%
OCD-c	5	3.66	2.10, 5.22	<0.0001	95%

### Meta-Regression Analyses

We conducted meta-regression analyses with regressed effect sizes on three potential moderators. Moderators include geographic region (Asia, Europe, and America), VR environments (non-immersive and immersive models), and comparators (OCD-control and Healthy control) to determine any moderation effects on primary outcomes. Meta-regression analyses showed that none of the selected moderators were statistically significant (Table 4).

**Table 4:** Meta-regression analysis for exploration of the sources of heterogeneity factors.

**Factors**	**Coefficient**	**Standard error**	**95% CI**	***p*-value**
Region	−1.07	0.98	−2.32, 0.84	0.25
Type of exposure	−0.29	0.82	−2.07, 1.57	0.69
Comparators	0.37	0.91	−1.35, 1.89	0.78

### Publication Bias

**Figure 4:**
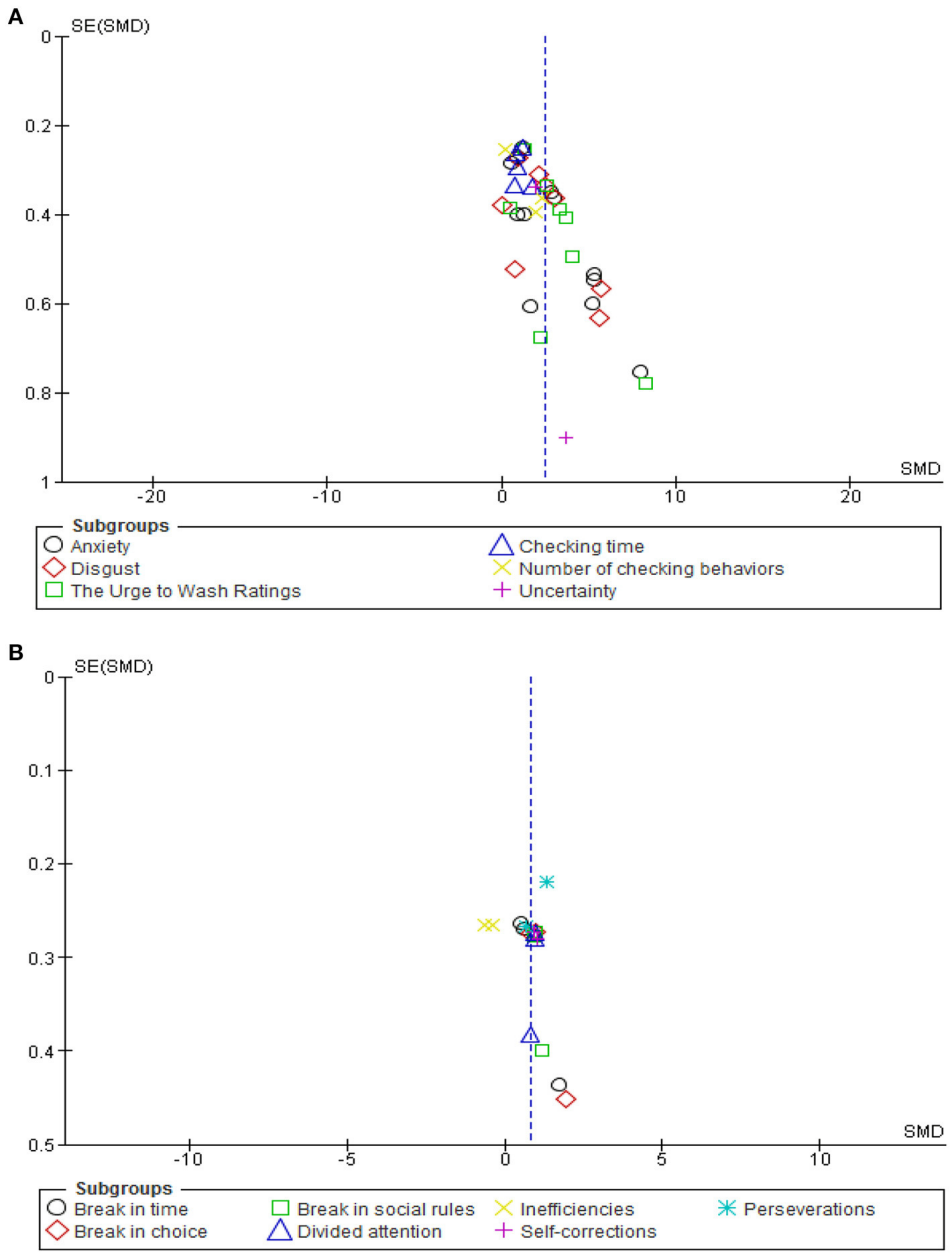
Funnel plot analysis for emotional response **(A)** to VR environment and VR scoring **(B)** in patients with OCD.

Funnel plots were used to find any risk of publication bias, whose shape was symmetrical. Moreover, performing Begg's test displays a non-significant asymmetry (*p* = 0.68). Therefore, we demonstrated no significant publication bias (Figure 4).

## Discussion

This systematic review and meta-analysis was performed to examine the effectiveness of VR-based technology on emotional response and symptoms in patients with OCD and found that this environment has a moderate enhancement in emotional response and symptoms test scores of patients with OCD. In this context, the effect of VR-based technology was previously evaluated and provided evidence supporting the positive impact of such technology on psychological disorders (50). Other research revealed that VR-based technology could significantly reduce the psychological symptoms (stress, anxiety, and depression) of malignancies (20, 51–53). Consequently, some research suggests that VR-based technology and relaxation techniques play an essential role in improving anxiety symptoms (54). On the other hand, it can be inferred that VR technology has a distracting effect from the rumination about their disease or negative cognition in depressed patients, which may be one reason for reducing depression in these patients. Besides, the amount of dopamine released in the brain increases with motor activity. In a wide range, this neurotransmitter causes a feeling of excitement and energy, happiness and vitality, and motivation that play a vital role in reducing depression (55, 56).

Our meta-analysis showed that the VR environment greatly provokes OCD symptoms, such as anxiety, disgust, checking time, and behaviors. In line with our findings, Kim et al. established a virtual environment to assess symptoms in OCD patients and found a greater provoked anxiety during both before and after checking a situation compared to healthy controls, and a higher decline in post-checking anxiety (17, 49). Furthermore, other researchers found a correlation of VR-based anxiety with the Y-BOCS and Beck's Anxiety Inventory scores (17, 57), and a higher checking frequency and checking time as well.

Despite substantial advances, there remain many challenges using new technology in patients with OCD (58). In contrast, some clinical studies provided evidence of higher levels of anxiety provocation and measurement following the use of VR-based technology in patients with OCD (15, 49). Our pooled analyses also showed a significant increase in emotional response and OCD symptoms when using VR-based technology. Higher emotional response, especially anxiety provocation caused by waiting for VR games, could be one of the possible reasons (15). Eventually, our findings on provoked emotions in response to VR-based technology in patients with OCD and OCD symptoms were in line with previous studies despite using different display techniques and software (18, 47, 49).

Moreover, we found a substantial increase in anxiety, urge to control, and uncertainty in patients with OCD after being exposed to a VR environment, which mostly supports the cognitive-behavioral analysis of reducing obsession-associated distress by performing compulsions in patients with OCD (59). In this context, van Bennekom et al. found a noteworthy decrease in both anxiety and urge to control symptoms. In contrast with our findings, they found no significant change in uncertainty after performing virtual compulsions in patients with OCD (15); however, no reduction in uncertainty could be due to the role of habit in compulsivity, with a shift of compulsions as targeted actions to habitual behavior during OCD (60).

Studies are currently concentrating on more complex psychiatric disorders, such as OCD, through assessing the anxiety-inducing potential of VR, with the goal of using exposure and response prevention. The main theory in clarifying the potential effects of VR is the Emotional Processing Theory (EPT), which is defined as the alteration of memory structures that underlie emotions (61). Another potential mechanism is inducing anxiety in the patients with OCD characterized by checking rituals. In this way, contributors were immersed with a head-mounted display in a virtual home or office where they had to turn on and off light switches, gas burners, and water faucets and, after a distraction phase, were invited to check what they did before leaving the virtual apartment (47, 49). A meta-analysis of VR-based interventions for anxiety, depression outcomes, and treatment attrition showed that this technology outperformed control conditions for anxiety and depression but did not improve treatment dropout (62).

Despite the provoking emotional response in some types of psychological disorders, VR-based technology may have incredible applications in the fields of medicine and mental health (63–65). Several research groups have been involved in this field in recent years, offering several treatments for panic, anxiety, neurological disorders, and even disabilities such as autism (66, 67). Therefore, VR-based technology was proposed as a promising treatment option in psychotherapy and will have many advantages in this field. Besides, many disadvantages have also been introduced, shedding light on potential adverse effects of VR-based technologies that should be considered seriously. First, disorientation or problem in determining the location; therefore, using VR-based technology devices in public and crowded places is potentially dangerous, especially in patients suffering from anxiety of being in crowded places or those who suffer from social anxiety (68). Another potential adverse event is vision problems after using VR-based technologies due to the effect on the binocular status of the eyes in the short term (69). However, research should be done more extensively and in more diverse samples to make the mechanism of these effects more visible.

VR characteristic can be more effective for future treatment designs for patients with OCD. Nevertheless, according to evidence from previous studies, the most predominant observations from patients were that they enjoyed the experience and loved the occasion to attempt a new technology. Scholars who desire to use VR technology in patients with OCD will be able to overcome undesirable adverse events, by regularly monitoring specific equipment, the scenarios, and the length of immersion to detect the best VR environment for each patient. As indicated, the VR technology is able to provoke core symptoms of anxiety and virtual compulsions in patients with OCD compared with healthy controls. Also, patients with OCD experienced a substantial drop in emotional responses after performing virtual compulsions. The situational factors involved in cognitive behavioral therapy (CBT), a type of treatment that helps individuals cope with and change problematic thoughts, behaviors, and emotions, are difficult to control, and often make it hard for psychotherapists to implement this therapy (70). So far, several CBTs have been put forward to develop and maintain OCD symptoms such as fear/anxiety and avoidance (71). Simultaneously, performing VR-based techniques such as imaginal exposure in association with CBT may have a positive effect on reducing anxiety and depression in patients with anxiety disorders such as OCD (72).

### Limitations

The present study has some limitations. Since the population of included studies is limited, generalizing the results to the whole population should be done with caution. Moreover, using standard scenarios is also another possible limitation. In the study, the VR setups that were designed for triggering contamination were not specific to the complaints or sensitivity of a specific individual. In addition, the analyses of the anxiety and disgust scores were evaluated with psychometric response scales like one-item visual analog scale (VAS) that has limitations in reliability and validity. Besides, the heterogeneous protocols used in terms of VR devices employed, program, and session durations could affect the results obtained in this review. Other significant limitations are the validity of various instruments and assessments used in the included studies, leading to bias. Information about patient gender was not clear and easy to collect in the present meta-analysis, which is an essential factor affecting the study findings.

## Future Perspective

Modulating emotional responses to virtual stimuli is an essential goal of several immersive interactive applications. It is important to know which VR characteristic is more effective in modulating the emotional responses of the patients with OCD. Thus, introducing VR technology with different immersive scenarios that aim to intensify emotional responses in patients with OCD may show promising outcomes (73). Remarkably, VRET decreases anxiety in phobic disorders (63), where a greater activation of emotions relates to positive treatment effects (74, 75). In line with recent studies (76), we encourage developing technologies that intensify the emotional response to VR stimuli, which ultimately might enhance treatment outcomes of VR interventions. VR-based studies on the association perception, emotional reaction such as fear, and VRET have shed light on the role of arousal as a basic dimension of emotional experience (77).

## Conclusion

VR-based technology was proposed as a new treatment with many advantages in medicine and mental health. Providing a direct patient-rated observation in the clinic, the VR-based technology permits therapists to evaluate detailed items triggering compulsions, the type and intensity of provoked emotions, and if compulsions provide relief or have to be repeated continuously without a reduction in emotions. Besides, many disadvantages have also been introduced, shedding light on potential adverse effects of VR-based technologies that should be considered seriously. This meta-analysis found that this environment has a moderate enhancement in emotional response and symptoms test scores of patients with OCD. However, our findings should be generalized with caution due to the lack of standardized methods and high heterogeneity among included evidence. The appropriate mode of integrating VR-based technology for patients with OCD requires more exploration. In future studies, it would be interesting to conduct future studies demonstrating the VR-based technology to recognize and diagnose OCD more quickly in primary care settings, as well as addressing the large gap between initiation of OCD symptoms and start of treatment.

## Data Availability Statement

The original contributions presented in the study are included in the article/Supplementary Material, further inquiries can be directed to the corresponding author/s.

## Author Contributions

FR, BD, SS, and MS were involved in the study design and drafting and editing of the manuscript. FR were involved in the experimental studies. FR, BD, and MS were involved in primer design, search, and data analysis. FR and SS were involved in revising the manuscript. All authors have read and approved the final manuscript.

## Conflict of Interest

The authors declare that the research was conducted in the absence of any commercial or financial relationships that could be construed as a potential conflict of interest.

## Publisher's Note

All claims expressed in this article are solely those of the authors and do not necessarily represent those of their affiliated organizations, or those of the publisher, the editors and the reviewers. Any product that may be evaluated in this article, or claim that may be made by its manufacturer, is not guaranteed or endorsed by the publisher.
