# Supplementary material for: The Effect of Virtual Reality on Emotional Response and Symptoms Provocation in Patients With OCD: A Systematic Review and Meta-Analysis
Source: Front Psychiatry. 2022 Feb 1;12:733584. doi: 10.3389/fpsyt.2021.733584 (PMC8846333; doi:10.3389/fpsyt.2021.733584)
Supplement: Supplementary file 1 [file Table_1.docx]

**The effect of virtual reality on emotional response and symptoms provocation in patients with OCD: A systematic review and meta-analysis**

**Supplementary files**

**Table 1.** PRISMA 2020 Checklist.

| **Section and Topic** | **Item #** | **Checklist item** | **Location where item is reported** |
| --- | --- | --- | --- |
| **TITLE** | | |  |
| Title | 1 | Identify the report as a systematic review. | 1 |
| **ABSTRACT** | | |  |
| Abstract | 2 | See the PRISMA 2020 for Abstracts checklist. | 2 |
| **INTRODUCTION** | | |  |
| Rationale | 3 | Describe the rationale for the review in the context of existing knowledge. | 5 |
| Objectives | 4 | Provide an explicit statement of the objective(s) or question(s) the review addresses. | 5 |
| **METHODS** | | |  |
| Eligibility criteria | 5 | Specify the inclusion and exclusion criteria for the review and how studies were grouped for the syntheses. | 6 |
| Information sources | 6 | Specify all databases, registers, websites, organisations, reference lists and other sources searched or consulted to identify studies. Specify the date when each source was last searched or consulted. | 6 |
| Search strategy | 7 | Present the full search strategies for all databases, registers and websites, including any filters and limits used. | 7 |
| Selection process | 8 | Specify the methods used to decide whether a study met the inclusion criteria of the review, including how many reviewers screened each record and each report retrieved, whether they worked independently, and if applicable, details of automation tools used in the process. | 7 |
| Data collection process | 9 | Specify the methods used to collect data from reports, including how many reviewers collected data from each report, whether they worked independently, any processes for obtaining or confirming data from study investigators, and if applicable, details of automation tools used in the process. | 7 |
| Data items | 10a | List and define all outcomes for which data were sought. Specify whether all results that were compatible with each outcome domain in each study were sought (e.g. for all measures, time points, analyses), and if not, the methods used to decide which results to collect. | 7 |
|  | 10b | List and define all other variables for which data were sought (e.g. participant and intervention characteristics, funding sources). Describe any assumptions made about any missing or unclear information. | 7 |
| Study risk of bias assessment | 11 | Specify the methods used to assess risk of bias in the included studies, including details of the tool(s) used, how many reviewers assessed each study and whether they worked independently, and if applicable, details of automation tools used in the process. | 8 |
| Effect measures | 12 | Specify for each outcome the effect measure(s) (e.g. risk ratio, mean difference) used in the synthesis or presentation of results. | 8 |
| Synthesis methods | 13a | Describe the processes used to decide which studies were eligible for each synthesis (e.g. tabulating the study intervention characteristics and comparing against the planned groups for each synthesis (item #5)). | 8 |
|  | 13b | Describe any methods required to prepare the data for presentation or synthesis, such as handling of missing summary statistics, or data conversions. | 8 |
|  | 13c | Describe any methods used to tabulate or visually display results of individual studies and syntheses. | 8 |
|  | 13d | Describe any methods used to synthesize results and provide a rationale for the choice(s). If meta-analysis was performed, describe the model(s), method(s) to identify the presence and extent of statistical heterogeneity, and software package(s) used. | 8 |
|  | 13e | Describe any methods used to explore possible causes of heterogeneity among study results (e.g. subgroup analysis, meta-regression). | 8 |
|  | 13f | Describe any sensitivity analyses conducted to assess robustness of the synthesized results. | 8 |
| Reporting bias assessment | 14 | Describe any methods used to assess risk of bias due to missing results in a synthesis (arising from reporting biases). | 8 |
| Certainty assessment | 15 | Describe any methods used to assess certainty (or confidence) in the body of evidence for an outcome. | 8 |
| **RESULTS** | | |  |
| Study selection | 16a | Describe the results of the search and selection process, from the number of records identified in the search to the number of studies included in the review, ideally using a flow diagram. | 9 |
|  | 16b | Cite studies that might appear to meet the inclusion criteria, but which were excluded, and explain why they were excluded. | 9 |
| Study characteristics | 17 | Cite each included study and present its characteristics. | 10 |
| Risk of bias in studies | 18 | Present assessments of risk of bias for each included study. | 11 |
| Results of individual studies | 19 | For all outcomes, present, for each study: (a) summary statistics for each group (where appropriate) and (b) an effect estimate and its precision (e.g. confidence/credible interval), ideally using structured tables or plots. | 12 |
| Results of syntheses | 20a | For each synthesis, briefly summarise the characteristics and risk of bias among contributing studies. | 13 |
|  | 20b | Present results of all statistical syntheses conducted. If meta-analysis was done, present for each the summary estimate and its precision (e.g. confidence/credible interval) and measures of statistical heterogeneity. If comparing groups, describe the direction of the effect. | 14 |
|  | 20c | Present results of all investigations of possible causes of heterogeneity among study results. | 15 |
|  | 20d | Present results of all sensitivity analyses conducted to assess the robustness of the synthesized results. | 16 |
| Reporting biases | 21 | Present assessments of risk of bias due to missing results (arising from reporting biases) for each synthesis assessed. | 17 |
| Certainty of evidence | 22 | Present assessments of certainty (or confidence) in the body of evidence for each outcome assessed. | 17 |
| **DISCUSSION** | | |  |
| Discussion | 23a | Provide a general interpretation of the results in the context of other evidence. | 19, 20 |
|  | 23b | Discuss any limitations of the evidence included in the review. | 21 |
|  | 23c | Discuss any limitations of the review processes used. | 21 |
|  | 23d | Discuss implications of the results for practice, policy, and future research. | 22 |
| **OTHER INFORMATION** | | |  |
| Registration and protocol | 24a | Provide registration information for the review, including register name and registration number, or state that the review was not registered. | - |
|  | 24b | Indicate where the review protocol can be accessed, or state that a protocol was not prepared. | - |
|  | 24c | Describe and explain any amendments to information provided at registration or in the protocol. | - |
| Support | 25 | Describe sources of financial or non-financial support for the review, and the role of the funders or sponsors in the review. | 23 |
| Competing interests | 26 | Declare any competing interests of review authors. | 24 |
| Availability of data, code and other materials | 27 | Report which of the following are publicly available and where they can be found: template data collection forms; data extracted from included studies; data used for all analyses; analytic code; any other materials used in the review. | 25 |

*Adapted From:*  McInnes MDF, Moher D, Thombs BD, McGrath TA, Bossuyt PM, The PRISMA-DTA Group (2018). Preferred Reporting Items for a Systematic Review and Meta-analysis of Diagnostic Test Accuracy Studies: The PRISMA-DTA Statement. JAMA. 2018 Jan 23;319(4):388-396. doi: 10.1001/jama.2017.19163.

For more information, visit: **www.prisma-statement.org**.

**Table S1.** Quality scoring for included five articles using Newcastle-Ottawa Scale (NOS) for case-control studies

Quality scoring for studies included in the meta-analysis. For each paper total ‘star’ (★) score was given consisting of; a letter (a, b or c) that stands for which of the NOS quality coding item list describe the paper more.

| **Author, year** | **Case**  **definition** | **Case**  **representation** | **Control**  **selection** | **Control**  **definition** | **Comparability** | **Exposure**  **ascertainment** | **Same method of**  **ascertainment** | **Response**  **rate** | **Total scores** |
| --- | --- | --- | --- | --- | --- | --- | --- | --- | --- |
|  |  |  |  |  |  |  |  |  |  |
| Inozu et al. 2021 (1) | a★ | a★ | c0 | a★ | a★ | 0 | a★ | 0 | 5 |
| van Bennekom et al. 2021 (2) | a★ | a★ | a★ | a★ | a★ | a★ | a★ | a★ | 8 |
| McCabe-Bennett et al. 2020 (3) | a★ | a★ | a★ | a★ | a★ | a★ | a★ | a★ | 8 |
| Jalal et al. 2020 (4) | a★ | a★ | a★ | a★ | 0 | a★ | 0 | 0 | 5 |
| Pedroli et al. 2019 (5) | a★ | a★ | a★ | a★ | a★ | a★ | a★ | a★ | 8 |
| van Bennekom et al. 2017 (6) | a★ | a★ | a★ | a★ | a★ | a★ | a★ | a★ | 8 |
| Laforest et al. 2016 (7) | a★ | a★ | c0 | a★ | a★ | a★ | a★ | a★ | 7 |
| LaPaglia et al. 2015 (8) | a★ | a★ | a★ | a★ | a★ | a★ | a★ | a★ | 8 |
| Cipresso et al. 2013(9) | a★ | a★ | a★ | a★ | a★ | a★ | a★ | a★ | 8 |
| Kim et al. 2012 (10) | a★ | a★ | c0 | a★ | a★ | a★ | a★ | a★ | 7 |
| Kim et al. 2008 (11) | a★ | a★ | a★ | a★ | a★ | a★ | a★ | a★ | 8 |

**NOS coding manual for case-control studies**

Selection

1) Is the Case Definition Adequate?

a) Yes, with independent validation ★ (e.g. >1 person/record/time/process to extract information, or reference to primary record source such as x-rays or medical/hospital records)

b) Yes, record linkage (e.g. ICD codes in database) or self-report with no reference to primary record

c) No description

2) Representativeness of the Cases

a) Consecutive or obviously representative series of cases ★

b) Potential for selection biases or not stated

3) Selection of Controls

a) Community controls ★ (i.e. same community as cases and would be cases if had outcome)

b) Hospital controls, within same community as cases (i.e. not another city) but derived from a hospitalized population

c) No description

4) Definition of Controls

a) No history of disease (endpoint) ★

b) No description of source

Comparability

1) Comparability of cases and controls on the basis of the design or analysis

A maximum of 2 stars can be allotted in this category

a) Study controls for the most important factor ★

b) Study controls for any additional factor ★ (This criteria could be modified to indicate specific control for a second important factor.)

Exposure

1) Ascertainment of exposure

a) Secure record (e.g. surgical records) ★

b) Structured interview where blind to case/control status ★

c) Interview not blinded to case/control status

d) Written self–report or medical record only

e) No description

2) Same method of ascertainment for cases and controls

a) Yes ★

b) No

3) Non-Response Rate

a) Same rate for both groups ★

b) Non respondents described

c) Rate different and no designation

1. Inozu M, Celikcan U, Trak E, Üzümcü E, Nergiz H. Assessment of Virtual Reality as an Anxiety and Disgust Provoking Tool: The Use of VR Exposure in Individuals With High Contamination Fear. Cyberpsychology. 2021;15.

2. van Bennekom MJ, de Koning PP, Gevonden MJ, Kasanmoentalib MS, Denys D. A Virtual Reality Game to Assess OCD Symptoms. Frontiers in psychiatry. 2020;11:550165.

3. McCabe-Bennett H, Lachman R, Girard TA, Antony MM. A Virtual Reality Study of the Relationships Between Hoarding, Clutter, and Claustrophobia. Cyberpsychology, behavior and social networking. 2020;23(2):83-9.

4. Jalal B, McNally RJ, Elias JA, Potluri S, Ramachandran VS. "Fake it till You Make it"! Contaminating Rubber Hands ("Multisensory Stimulation Therapy") to Treat Obsessive-Compulsive Disorder. Frontiers in human neuroscience. 2019;13:414.

5. Pedroli E, La Paglia F, Cipresso P, La Cascia C, Riva G, La Barbera D. A Computational Approach for the Assessment of Executive Functions in Patients with Obsessive-Compulsive Disorder. Journal of clinical medicine. 2019;8(11).

6. van Bennekom MJ, Kasanmoentalib MS, de Koning PP, Denys D. A Virtual Reality Game to Assess Obsessive-Compulsive Disorder. Cyberpsychology, behavior and social networking. 2017;20(11):718-22.

7. Laforest M, Bouchard S, Cretu A-M, Mesly O. Inducing an Anxiety Response Using a Contaminated Virtual Environment: Validation of a Therapeutic Tool for Obsessive–Compulsive Disorder. Frontiers in ICT. 2016;3.

8. la Paglia F, la Cascia C, Rizzo R, Riva G, la Barbera D. Decision Making and Cognitive Behavioral Flexibility in a OCD Sample: a Study in a Virtual Environment. Studies in health technology and informatics. 2015;219:53-7.

9. Cipresso P, La Paglia F, La Cascia C, Riva G, Albani G, La Barbera D. Break in volition: a virtual reality study in patients with obsessive-compulsive disorder. Experimental brain research. 2013;229(3):443-9.

10. Kim K, Roh D, Kim CH, Cha KR, Rosenthal MZ, Kim SI. Comparison of checking behavior in adults with or without checking symptom of obsessive-compulsive disorder using a novel computer-based measure. Computer methods and programs in biomedicine. 2012;108(1):434-41.

11. Kim K, Kim CH, Cha KR, Park J, Han K, Kim YK, et al. Anxiety provocation and measurement using virtual reality in patients with obsessive-compulsive disorder. Cyberpsychology & behavior : the impact of the Internet, multimedia and virtual reality on behavior and society. 2008;11(6):637-41.

**Figure S1.** Forest plot of comparison of anxiety between various geographic regions across included studies.


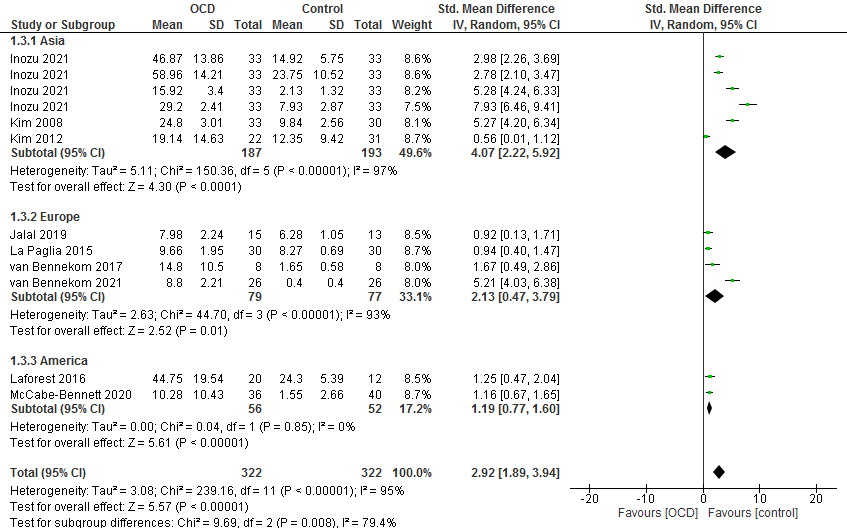


**Figure S2.** Funnel plot of comparison of anxiety between various geographic regions across included studies.

**
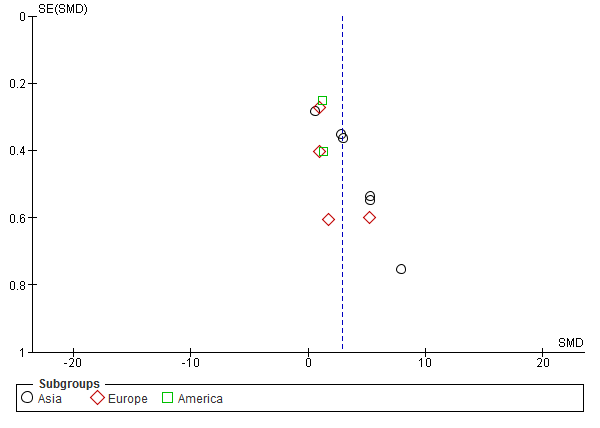
**

**Figure S3.** Forest plot of anxiety between various comparators across included studies.

**
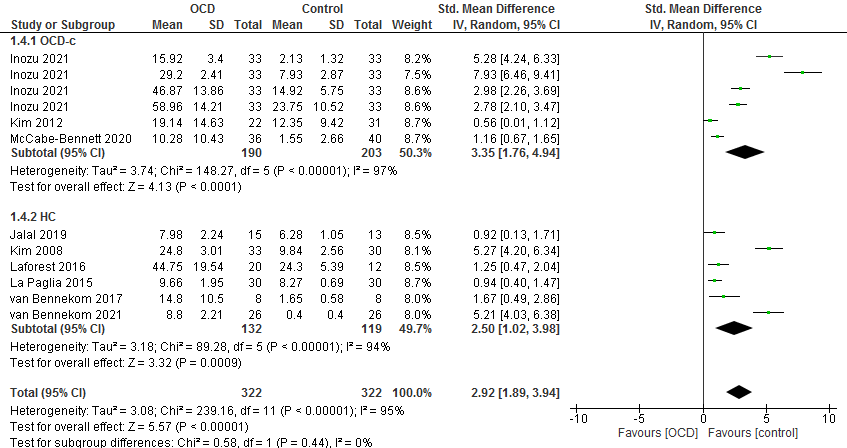
**

**Figure S4.** Funnel plot of anxiety between various comparators across included studies.

**
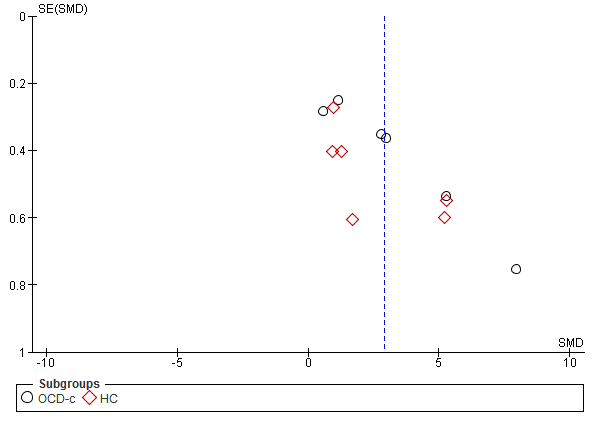
**

**Figure S5.** Forest plot of anxiety between various types of VR technologies across included studies (VRE, VR environment).


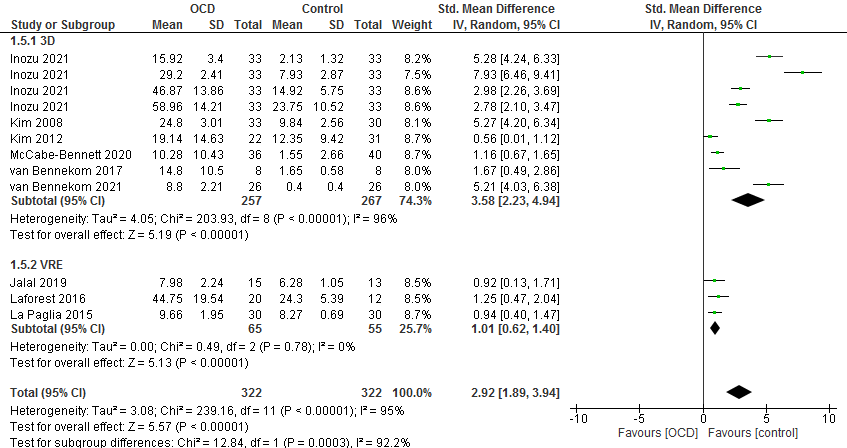


**Figure S6.** Funnel plot of anxiety between various types of VR technologies across included studies (VRE, VR environment).

**
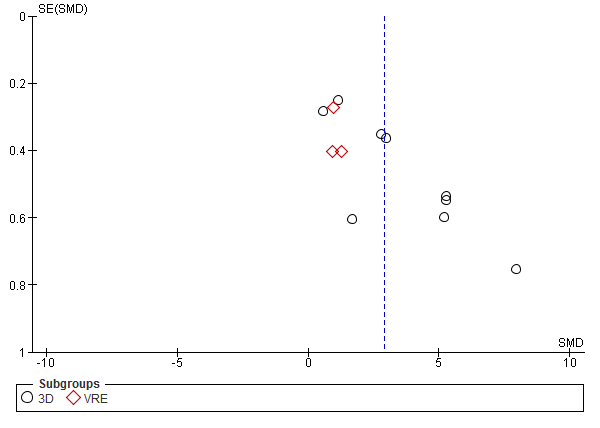
**

**Figure S7.** Forest plot of comparison of disgust between various geographic regions across included studies.

**
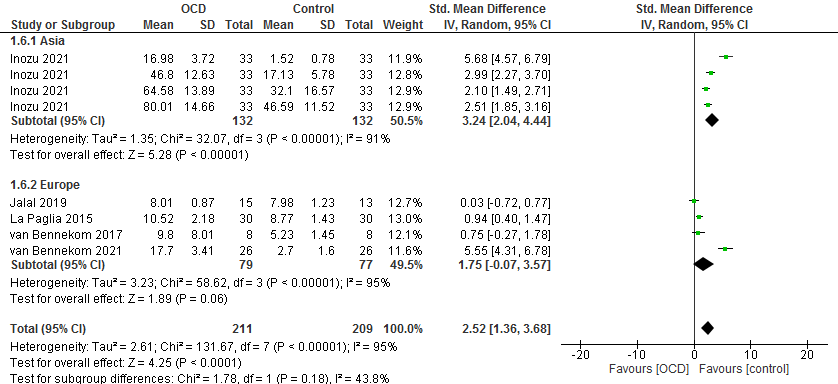
**

**Figure S8.** Funnel plot of comparison of disgust between various geographic regions across included studies.

**
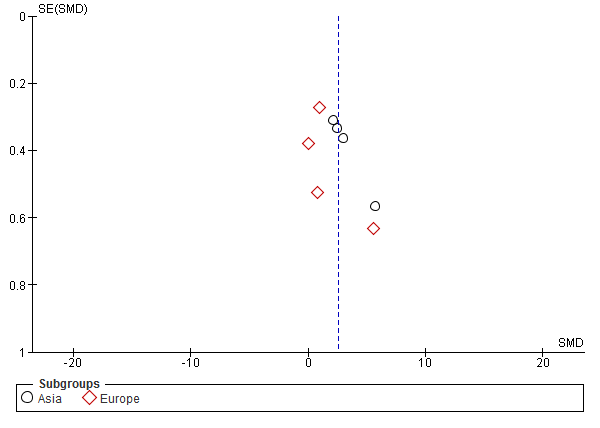
**

**Figure S9.** Forest plot of disgust between various comparators across included studies.

**
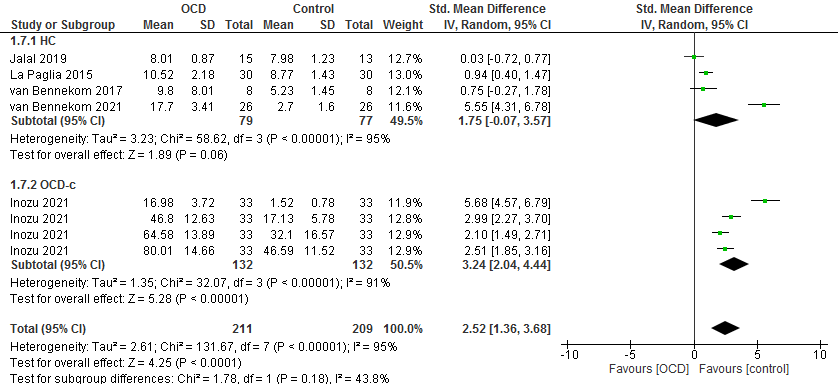
**

**Figure S10.** Funnel plot of disgust between various comparators across included studies.

**
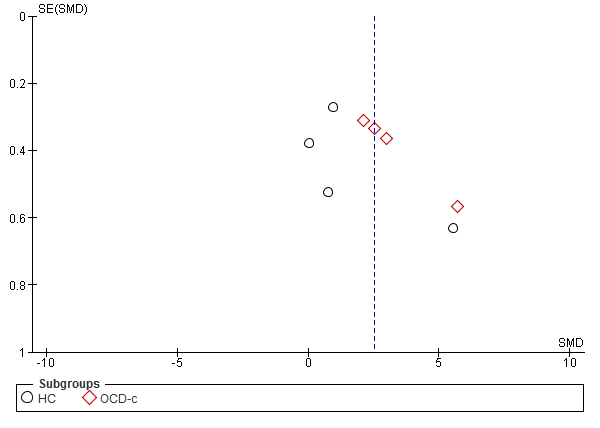
**

**Figure S11.** Forest plot of disgust between various types of VR technologies across included studies (VRE, VR environment).


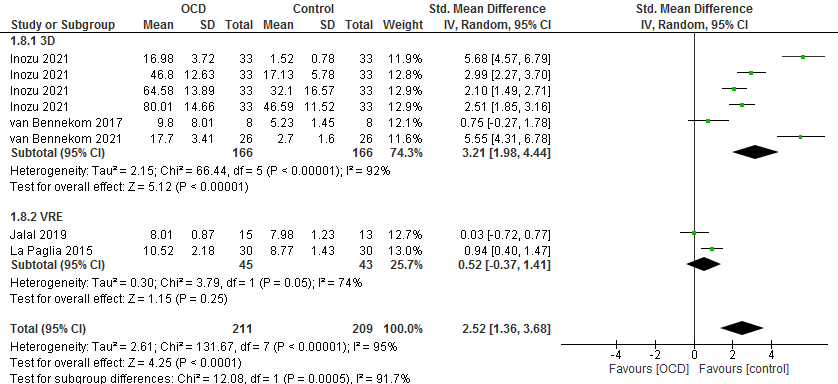


**Figure S12.** Funnel plot of disgust between various types of VR technologies across included studies (VRE, VR environment).


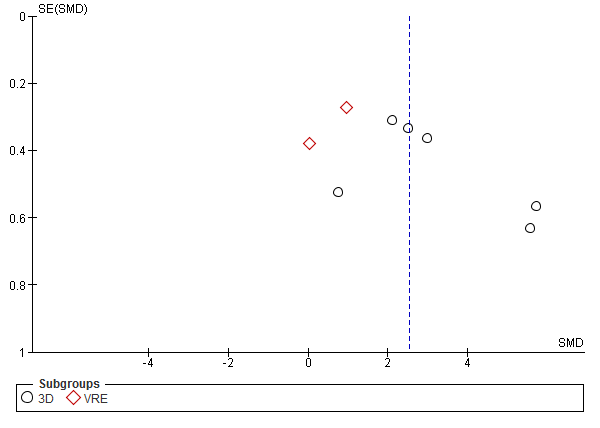


**Figure S13.** Forest plot of comparison of urge to wash between various geographic regions across included studies.

**
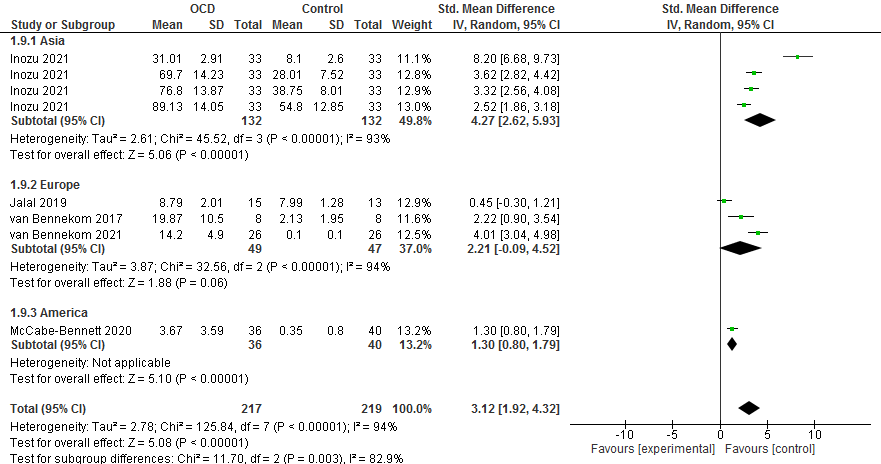
**

**Figure S14.** Funnel plot of comparison of urge to wash between various geographic regions across included studies.

**
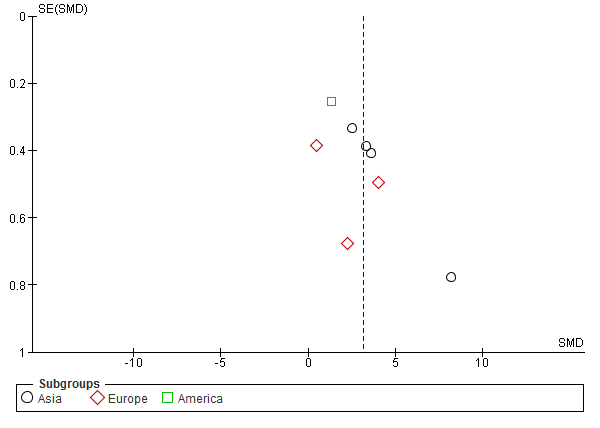
**

**Figure S15.** Forest plot of urge to wash between various comparators across included studies.

**
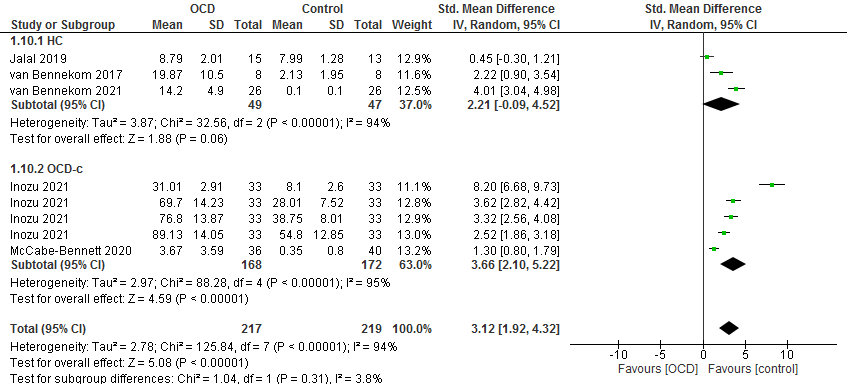
**

**Figure S16.** Funnel plot of urge to wash between various comparators across included studies.

**
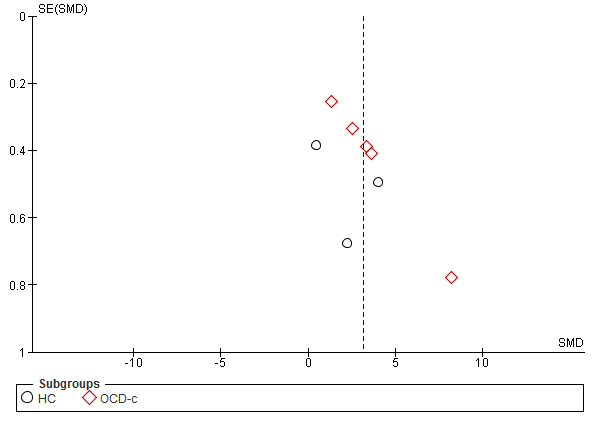
**

**Figure S17.** Forest plot of urge to wash between various types of VR technologies across included studies (VRE, VR environment).

**
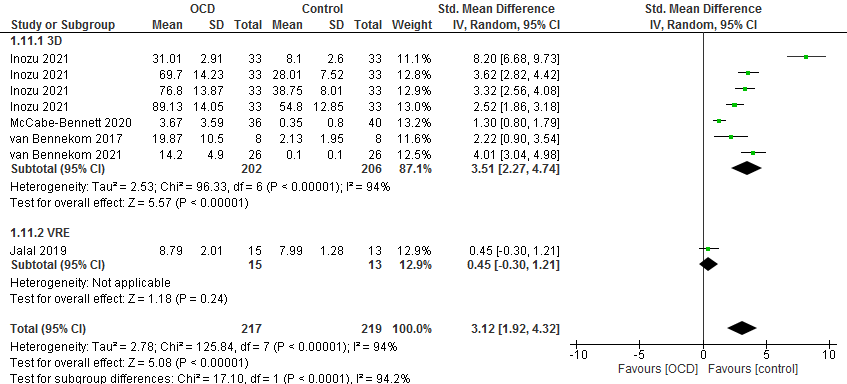
**

**Figure S12.** Funnel plot of urge to wash between various types of VR technologies across included studies (VRE, VR environment).

**
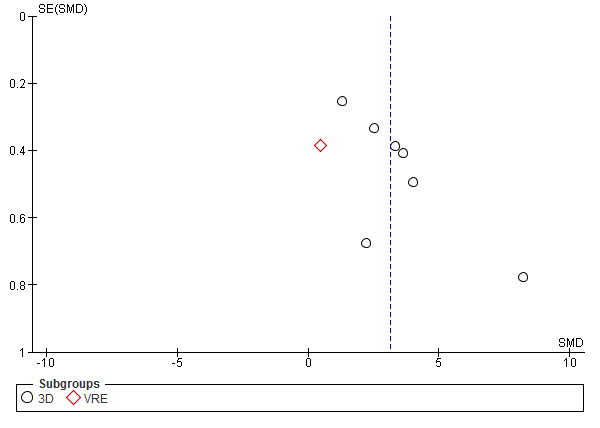
**
